# Gene expression in acute Stanford type A dissection: a comparative microarray study

**DOI:** 10.1186/1479-5876-4-29

**Published:** 2006-07-06

**Authors:** Barbara Theresia Weis-Müller, Olga Modlich, Irina Drobinskaya, Derya Unay, Rita Huber, Hans Bojar, Jochen D Schipke, Peter Feindt, Emmeran Gams, Wolfram Müller, Timm Goecke, Wilhelm Sandmann

**Affiliations:** 1 Department of Vascular Surgery and Kidney Transplantation, Heinrich-Heine-University of Düsseldorf, Düsseldorf, Germany; 2 Department of Chemical Oncology, Heinrich-Heine-University of Düsseldorf, Düsseldorf, Germany; 3 Research Group Experimental Surgery, Heinrich-Heine-University of Düsseldorf, Düsseldorf, Germany; 4 Department of Thoracic and Cardiovascular Surgery, Heinrich-Heine-University of Düsseldorf, Düsseldorf, Germany; 5 Pathology Starnberg, private pathological practice, Starnberg, Germany; 6 Institute of Human Genetics, Heinrich-Heine-University of Düsseldorf, Düsseldorf, Germany

## Abstract

**Background:**

We compared gene expression profiles in acutely dissected aorta with those in normal control aorta.

**Materials and methods:**

Ascending aorta specimen from patients with an acute Stanford A-dissection were taken during surgery and compared with those from normal ascending aorta from multiorgan donors using the *BD Atlas™ Human1.2 Array I*, *BD Atlas™ Human Cardiovascular Array *and the *Affymetrix HG-U133A GeneChip*^®^. For analysis only genes with strong signals of more than 70 percent of the mean signal of all spots on the array were accepted as being expressed. Quantitative real-time polymerase chain reaction (RT-PCR) was used to confirm regulation of expression of a subset of 24 genes known to be involved in aortic structure and function.

**Results:**

According to our definition expression profiling of aorta tissue specimens revealed an expression of 19.1% to 23.5% of the genes listed on the arrays. Of those 15.7% to 28.9% were differently expressed in dissected and control aorta specimens. Several genes that encode for extracellular matrix components such as collagen IV α2 and -α5, collagen VI α3, collagen XIV α1, collagen XVIII α1 and elastin were down-regulated in aortic dissection, whereas levels of matrix metalloproteinases-11, -14 and -19 were increased. Some genes coding for cell to cell adhesion, cell to matrix signaling (*e.g*., polycystin1 and -2), cytoskeleton, as well as several myofibrillar genes (*e.g*., α-actinin, tropomyosin, gelsolin) were found to be down-regulated. Not surprisingly, some genes associated with chronic inflammation such as interleukin -2, -6 and -8, were up-regulated in dissection.

**Conclusion:**

Our results demonstrate the complexity of the dissecting process on a molecular level. Genes coding for the integrity and strength of the aortic wall were down-regulated whereas components of inflammatory response were up-regulated. Altered patterns of gene expression indicate a pre-existing structural failure, which is probably a consequence of insufficient remodeling of the aortic wall resulting in further aortic dissection.

## Background

Aortic dissection is a life threatening disease developing without any warning. Modern diagnostic methods, such as computed or magnetic resonance tomography, are able to show an aortic wall hematoma at the acute onset of the disease. This hematoma develops to aortic wall dissection over time. It is proposed that bleeding of the *vasa vasorum *presents the first step of dissection [1,2]. Subsequently intimal tears in the aortic wall will develop later forming a connection within the aortic wall to the aortic blood stream, which at the end results in two blood stream channels, the true and false aortic lumen.

Little is known about the underlying defects of aortic dissection. Patients with heritable connective disorders, such as Marfan-syndrome patients with a defect of the glycoprotein fibrillin-1, and Ehlers-Danlos-syndrome patients with a type III-procollagen disorder are known to develop aortic dissection very early in their life [3-10]. However, only few patients with aortic dissection suffer from one of these syndromes as found in 134 Stanford A- and 158 Stanford B patients treated at our institution between 1984 and 2002. No patients with Ehlers-Danlos syndrome were found and only 9 patients (3.1%) of this cohort fulfilled all criteria of a Marfan-syndrome. Nonetheless, it is notable that some of the dissection patients resembled Marfan patients in some aspects and demonstrated for example joint hypermobility or skin abnormalities [11]. Based on these observations the question arises: "Does an unknown connective tissue disorder cause a predisposition for aortic dissection?"

In order to answer this question, in 2002 we performed our first comparative molecular study of acutely dissected human aorta and normal control aorta based on measurements of gene expression [12]. This previous study was considered a pilot study with no quantitative real-time polymerase chain reaction (RT-PCR) being regarded as the gold standard to validate microarray data [13]. Following the editor's recommendation to pursue the study, in the given article we report the second expanded validation study on a molecular basis of acute dissected aortic disease. Now, we focussed on specific cardiovascular genes and compared the results of two different membrane-based cDNA arrays (Clontech platform), GeneChip oligonucleotide microarray (Affymetrix platform) and real-time RT-PCR.

## Patients, materials and methods

### Patients' samples

For the Atlas array experiments, ascending aorta specimen were obtained from 8 patients operated on for acute Stanford type A aortic dissection (6 men 40, 41, 41, 49, 52, 54 years old and 2 women 52 and 63 years old; mean 57.5 ± 11.7 years) and from 8 multi organ donors (3 men 23, 42, 65 years old and 5 women 40, 42, 43, 45, 53 years old; mean 44.0 ± 11.9 years). For the Affymetrix array experiments, ascending aorta specimen were taken from 4 Stanford type A patients (4 men 40, 41, 52, 54 years old; mean 46.8 ± 7.3 years) and from 4 multiorgan donors (2 man 65 and 40 years old; mean 53 ± 14, and 2 women 42, 45 years old; mean 42.5 ± 2.8 years). Patients' demographics as well as specification in what experiment samples have been analyzed are shown in Table 1. Informed written consent was obtained from the patients or their relatives. Samples were "snap-frozen" in liquid nitrogen and were stored at -80°C. All studies were conducted under protocols approved by the University Review Board and the local Ethical Committee.

**Table 1:** Patients' demographics and usage in microarray experiments

Case ID time	Sex	Age, years	Aorta sample	Human 1.2 array I	Human Cardiovascular array	Affymetrix HG-133A	Real-PCR
p1	m	56	Stanford A	used	used	used	used
p2	m	41	Stanford A	used	used	used	used
p3	m	40	Stanford A	used	used	used	used
p4	f	52	Stanford A	used	used	no	no
p5	f	63	Stanford A	used	used	no	used
p6	m	41	Stanford A	used	used	no	used
p7	m	54	Stanford A	used	used	no	used
p8	m	52	Stanford A	used	used	used	used
p9	f	58	Stanford A	no	no	no	used
p10	m	60	Stanford A	no	no	no	used
c1	m	65	Control	used	used	used	used
c2	f	45	Control	used	used	used	used
c3	m	40	Control	used	used	used	used
c4	f	44	Control	used	used	used	used
c5	m	23	Control	used	used	no	used
c6	f	42	Control	used	used	no	used
c7	m	42	Control	used	used	no	used
c8	f	54	Control	used	used	no	no

### Total RNA isolation

Aorta tissue specimens were pulverized under liquid nitrogen, and total RNA was extracted from grinned tissue samples by cell lysis and subsequent binding to RNAeasy mini spin columns including DNase I treatment (Qiagen GmbH, Hilden, Germany). Subsequent washing and elution steps were performed according to the manufacturer's protocol. Total RNA was quantified using UV spectrophotometry (Photometer ECOM 6122, Eppendorf AG, Hamburg, Germany) and the quality and integrity was confirmed by electrophoresis of 0.5 μg isolated RNA on 1% formamide agarose gels. Additionally, RNA specimens were analyzed by microcapillary electrophoresis on LabChips using the Agilent 2100 bioanalyzer (Agilent Technologies GmbH, Boeblingen, Germany) following the manufacturer's instructions.

### Microarray experiments

Three different gene expression studies were carried out. For the first gene expression study, we used the *BD Atlas™ Human1.2 Array I *(Biosciences, Clontech Laboratories, Heidelberg, Germany) with 1,185 single spotted probes for known human genes. This set of genes was completed by a second study with the *BD Atlas™ Human Cardiovascular Array *(BD Biosciences, Clontech Laboratories), which contains 597 double spotted probes for genes that are specific for the cardiovascular system. Both arrays are membrane-based and need to be probed with radioactive labeled cDNAs. There are 138 genes in common between the two Atlas arrays. All samples applied with Human 1.2 arrays were used in experiments with Cardiovascular arrays. For genome wide screening of differentially expressed genes, a third gene expression study was performed using the *Affymetrix Human Genome U133A GeneChip*^® ^with 22,283 features for human genes/ESTs. For hybridization and analysis, biotin labeled cRNA (4 dissected and 4 control samples, Table 1) was used.

### Atlas experiments, cDNA probe synthesis

Radiolabeled cDNAs were prepared from 5 μg of total RNA by incorporating 30 μCi [α-^32^P] dATP into cDNA by reverse transcription using a gene specific primer mix (BD Biosciences Clontech) as described [12]. Incorporation rates were ~10%. Filter arrays (Atlas™ Human Broad I and Cardiovascular) were pre-hybridized ~1 h in a hybridization buffer (ExpressHyb, Clontech) and membranes were hybridized for 16–18 h at 68°C. After extensive washes membranes were exposed to a phosphor-imaging screen (Fuji Raytest, Inc.) for 48 h. Stripped and QC-checked membranes were reused for the next probe hybridization for maximum three hybridizations. Reproducibility of experiments was verified by comparing one hybridization experiment with the same or newly prepared probe on two or, in some cases, four different array membranes. The results showed good reproducibility within independent hybridizations with correlation coefficients between 0.90 and 0.98.

### Atlas experiments, data analysis

Primary data collection and analysis were carried out using Phosphorimager (BAS-1500, Fuji, Raytest, Straubenhardt, Germany) and Aida software (Raytest). Thus, the amount of radioactivity on the membrane was measured by means of a phosphorimager and converted into corresponding gray levels of an image by the corresponding software. The gray levels are supposed to be linearly correlated to the amount of the radioactivity on the filter. The linearity has been experimentally checked by comparison of measurements on a particular array during different exposure times (8, 16, 24, 32, and 48 h of the exposure of a filter to the phosphorimager screen). The 24 h exposure has been found appropriate to avoid the oversaturation of the radioactive spots corresponding to the most intensive signals. For each sample, we measured the pixel intensity of the 597 genes spotted in duplicates on single filter (Atlas Human Cardiovascular Array) or for 1,185 single-spotted probes (Atlas Human Total I) on two different filters. Radioactive intensity of each spot on the membrane was linearly digitized to gray scale with a pixel size of 100 μm. Data were obtained as a list of intensity values for all measured positions on the array. Areas of arrays with obviously overlapping signals and artifacts were manually excluded from further analyses. The background signal was subtracted using Aida software and average pixel intensity for each pair of spots (or for each spot) was generated.

Normalization of signal intensities obtained from different hybridization experiments was based on the sum of background-subtracted signal data of all expressed genes (total intensity normalization). The total intensity normalization is one of wildly used techniques that can be used to normalize gene expression values from a single microarray [14]. This step was performed to account for differences in labeling and quality of RNA samples. Finally, mean values of intensity for each gene detected from multiple arrays were generated by the computer software as an average array.

Additionally, normalization using ubiquitin, cytoplasmic beta-actin (*ACTB*) and 60S ribosomal protein L13A (*RPL13A*) housekeeping genes' expression values has been applied to raw gene expression data and results were compared with those from the mean value normalization. The resulting table containing data for both normalization methods is available on the Website Cancer today [43].

### Affymetrix human genome U133A GeneChip

The preparation and processing of labeled and fragmented cRNA targets as well as GeneChip hybridization and scanning was carried out according to the manufacturer's protocol (Affymetrix, Santa Clara, CA, USA) as described elsewhere [15]. Starting material for the labeling procedure was 5 μg of total RNA from each aortic specimen, and the labeling procedure was limited to one cycle of *in vitro *transcription. Synthesized and fragmented cRNAs were analyzed by electrophoresis using LabChips on Agilent 2100 Bioanalyzer. After washing and staining steps, arrays were scanned with Gene Array scanner 2500 (Affymetrix). Hybridization intensity data were automatically acquired and processed by Affymetrix Microarray Suite 5.0 software. The expression level (average difference) for each gene was determined by calculating the average of differences in intensity (perfect match-mismatch) between its probe pairs as described elsewhere [16]. In order to normalize for sample loading and staining variation, the average fluorescent intensities of all hybridization probes on an individual array were scaled to fixed target signal intensity TGT of 100 relative signal units (RSU).

The study reported in our manuscript complies with the Minimum Information About a Microarray Experiment (MIAME) guidelines established by the Microarray gene expression data society .

### Quantitative Real-Time PCR (RT-PCR)

We followed one of the most striking recent developments in the confirmatory studies issue [13] and authors are informed about the latest discussion concerning establishing standards for microarray data annotation and representation [17]. In order to validate an array results for relevance, RT-PCR has been used.

Aliquots of the same total RNA as for microarray analysis were used for quantitative RT-PCR. Validation of arrays data has been performed for 24 differentially expressed genes. All analyses were performed using the ABI PRISM 7900 Sequence Detection System (Applied Biosystems, Foster City, CA, USA).

cDNAs for all PCR amplifications were generated by oligo-dT primed reverse transcription (Superscript First Strand System, Invitrogen Corporation, Carlsbad, CA, USA). Primers and probes were designed by the manufacturer (Assays-on-Demand; Applied Biosystems, Foster City, CA, USA). *RPLPO *(Hs 9999902m1) and cyclophilin A (Hs 99999904m1) were selected as reference genes to standardize the amount of sample RNA. The respective primer/probes were prepared by mixing 25 μl of the 100 μM stock solution "upper primer", 25 μl of the 100 μM stock solution "lower primer" with 12,5 μl of the 100 μM stock solution TaqMan-probe (FAM/TAMRA) and adjusted to 500 μl with H_2_0 (primer/probe-mix). PCR reactions using cDNA generated from 20 ng of total RNA were performed in duplicates in a volume of 25 μl. This includes TaqMan universal Master Mix (Applied Biosystems) according to the manufacturer's protocols in 96-well format and 1.25 μl of the Probe&Primer mix. Thermal cycler parameters were 2 min at 50°C, 10 min at 95°C and 40 cycles, each consisting of a 15 s denaturation step at 95°C and a 1 min annealing/extension step at 60°C. Relative abundance of a gene transcript was calculated by the ΔΔCt method.

### Statistical analyses

#### Student's t-test

Mean values were calculated for each gene from the control and dissected cohorts of samples. Weak signals on the array close to background result usually in unfavorable signal-to-noise ratio and show low reproducibility between experiments. Ratios of differential gene expression based on such values may be very high even when there is no significant difference in the expression level of the corresponding genes [14]. Therefore, only genes with a mean expression value of at least 70% of the mean expression of all genes spotted on the array, either in the control or in the dissected aorta group, were used for subsequent analyses. Genes were estimated as "differentially expressed" in dissected and control aorta, if the mean expression ratio of dissected and control aorta was less than 0.6 or more than 1.8 for Human and Cardiovascular filter arrays. For Affymetrix data, the same cut-off for mean expression value of at least 70% of expression of all genes per array has been applied. Since Affymetrix platform is more precise and sensitive in detecting of signals corresponding expressed mRNAs than arrays using radiolabeled probes minimal fold change was set to 2.0 for Affymetrix data. The unpaired Student's *t*-test was used as appropriate with significance set at a *P *level of < 0.05 to compare gene expression values for all three arrays. All *t*-tests were two-sided.

#### Identification of differentially expressed genes using "Significance Analysis of Microarrays" (SAM)

Additionally, genes differentially expressed between dissected and control aorta were identified using the significance analysis of microarrays (SAM) software. The SAM software is a statistical tool for finding significant genes in a set of microarray experiments. Idea was proposed by Tusher, Tibshirani and Chu [18]. This software was written by B. Narasimhan and R. Tibshirani [43]. It is freely available for academic organizations. It works as a Microsoft Excel add-in. Control and dissected aorta datasets were separated to corresponding blocks as recommended by SAM procedure. Readers are referenced to the SAM online manual for detailed explanations of the approach. SAM applies some test statistic, for example, a t-statistic, and then uses permutation to draw inferences. Shortly, SAM calculates a statistic *d*_*i *_for each gene *i *by measuring the strength of the relationship between gene expression and the class variable (control and dissected aorta). SAM uses repeated permutations of the data to determine the significant gene expression relating to the specific class. The cutoff for significance is determined by a tuning parameter *delta*. *Delta *is the difference between the observed score (*t*-statistics for the true labels), and the average expected score (*t*-statistics for the randomly permutated data). *Delta *is chosen by user based on the false discovery rate (FDR). A *fold change *parameter can be chosen to ensure the pre-specified amount of differential gene expression. *Fold change *of gene expression is the ratio of the mean expression levels for this gene in the groups under comparison. User definable conditions were set to default values. We applied two class unpaired comparison with *t*-statistics. Gene expression data were unlogged. Value of *k *parameter in *k*-nearest-neighbor algorithm was set to 3, number of permutations was 500. In these studies the value of *delta *was always chosen so that estimated FDR was a zero.

#### Partial least squares discriminant analysis for multivariate data (PLS-DA)

We applied a direct linear discriminant analysis to compare expression profiles of control and dissected aorta. Supervised PLS-DA uses independent (expression levels) and dependent variables (classes) for class comparison applying multivariate statistical methods, namely the soft independent modeling of class analogy (SIMCA) and partial least squares modeling with latent variables to allow simultaneous analysis of all variables [19-22]. Additionally, PLS-DA provides a quantitative estimation of the discriminatory power of each descriptor (gene) by means of *VIP *(variable importance of the projection) parameters. *VIP *values rank descriptors according to their ability to discriminate different classes. PLS-DA was performed with SIMCA-P commercially software (Umetrics AB, Umea, Sweden) as described by Pérez-Enciso and Tenenhaus [22]. Model validation was carried out via permutation. Number of permutations was 100. Parameter *Q*^2 ^is a measure of the predictive ability of the model, and *R*^2 ^is related to the goodness of fit of the model.

Although the application of the *t *test for selection of genes is appropriate because the number of defined classes is limited to two [20], the *VIP *criterion used in PLD-DA is more robust and discriminative. The PLS-DA is not hampered by the problem of data that is not distributed per normality; an assumption that has to be made for the standard parametric *t *test. This assumption is rather more important when working with relatively small numbers of samples as in the present study.

## Results

### Identification of genes differentially expressed in dissected and control aorta, microarray analysis by different statistical methods

#### a. Unpaired t-test

##### BD Atlas™ human1.2 array

According to our definition (see section "Statistical analyses" in Material and Methods) 229 of 1,185 genes (19.3%) were expressed. Of those, 49 genes (21.4%) were differentially expressed. Twenty-five genes showed higher and 24 lower levels of gene expression in the dissected compared with the control aorta.

##### BD Atlas™ human cardiovascular array

114 of 597 genes (19.1%) were estimated as being expressed. Thirty-three genes (28.9%) displayed differential expression, 10 genes were up-regulated, whereas 23 genes were down-regulated in the dissected aorta.

##### Affymetrix HG-133A GeneChip

On the Affymetrix platform 5246 of 22,283 genes (23.5%) showed detectable expression, of which 823 genes (15.7%) were differentially expressed in dissected and control aorta. Of those, 323 genes showed higher and 500 genes lower expression in dissected aorta.

In table 2 all differently expressed genes of the Clontech Atlas experiment are shown and compared to the Affymetrix experiment classified to several functional groups in the similar way how it has been done in our first published study [12]. Additionally to the differentially expressed genes as observed by use of both Clontech Atlas filter arrays, we included several significantly different expressed genes of the Affymetrix experiment in order to show functional groups to make it more informative for common readers.

**Table 2:** Gene expression studies: dissected aorta ascendens vs. control aorta. Gene expression studies that comparing freshly dissected aorta ascendens (D) and undissected control aorta (C). 3 different gene expression studies were performed: 16 samples were studied with *BD Atlas™ Human1.2 Array *(h), 16 samples – with *BD Atlas™ Human Cardiovascular Array *(c) and 8 samples – with *Affymetrix Human Genome U133A GeneChip*. All differently expressed genes of the *Atlas*-experiments, 49 genes (h) and 33 genes (c), are shown and compared to genes of the *Affymetrix *experiment. Ratio: mean expression of dissected aorta (D) divided by mean expression of control (C); n.e.: not expressed, n.s.: difference not significant. n.t.: not tested, ↓ downregulated, ↑ upregulated; ? – regulation in the Atlas and Affymetrix experiment different

**Gene Name**	**Platform**	**Atlas**		**Affymetrix**		**regulation**
**Functional group**	**Array type**	**ratio D/C**	***P *value**	**ratio D/C**	***P *value**	
**extracellular matrix proteins,**						
**cell surface antigens, adhesion proteins**						
collagen IV α2, COL4A2	h	0.5	<0.01	0.4	<0.05	↓
collagen IV α5, COL4A5		n.t.		0.32	<0.05	↓
collagen VI α3, COL6A3	c	1.9	<0.05		n.s.	**?**
collagen VIII α1, COL8A1	c	0.3	<0.01	0.3	<0.001	↓
collagen VIII α2, COL8A2		n.t.		0,4	<0,01	↓
collagen XIV α1, COL14A1, undulin	c	0.4	<0.001	0.5	<0.001	↓
collagen XVIII α1, COL18A1		n.t.		0.47	<0.001	↓
polycystin1, PKD1	h	0.3	<0.001	0.4	<0.01	↓
polycystin 2, PKD2		n.t.		0.4	<0.001	↓
clusterin, SP-40;	h	0.5	<0.01	0.5	<0.01	↓
integrin α7B, IGA7B	h	0.4	<0.001	0.3	<0.001	↓
gap junction protein, connexin 43	c	0.6	<0.05		n.s.	**?**
SPARC-like 1	c	0.3	<0.001	0.6	<0.001	↓
decorin	c	0.3	<0.05		n.s.	↓
elastin		n.t.		0.4	<0.05	↓
fibulin 5		n.t.		0.4	<0.001	↓
cell surface glycoprotein MUC18	h	0.3	<0.001	0.5	<0.01	↓
fibronectin receptor	h	n.s.		3.4	<0.01	↑
ras homolog gene family member B	h	0.5	<0.05		n.s.	**?**
**extracellular matrix proteolysis**						
MMP-11	h	2.1	<0.001	3.2	<0.05	↑
MMP-14		n.t.		2.5	<0.01	↑
MMP-19		n.t.		3.2	<0.05	↑
TIMP1	h,c	1.8	<0.01		n.s.	**?**
TIMP2	h,c	0.4	<0.01		n.s.	**?**
TIMP3	c	0.2	<0.001		n.s.	**?**
**cell motility and cytoskeleton proteins**						
vinculin	c	0.2	<0.001		n.s.	**?**
actinin α1	c	0.5	<0.001	0.8	<0.01	↓
filamin A	c	0.3	<0.001	0.5	<0.001	↓
actin α2		n.t.		0.13	<0.001	↓
actinin α4		n.t.		0.44	<0.001	↓
gelsolin		n.t.		0.5	<0.01	↓
leiomodin		n.t.		0.4	<0.01	↓
tropomyosin 1		n.t.		0.4	<0.01	↓
calponin		n.t.		0.3	<0.001	↓
smooth & non-muscle myosin light chain kinase	h	0.4	<0.001	n.t.		↓
smooth muscle myosin heavy chain, SM2		n.t.		0.5	<0.001	↓
myosin regulatory light chain2, smooth muscle		n.t.		0.5	<0.001	↓
**metabolism**		n.t.				
low-density lipoprotein receptor, LDLR	c	0.4	<0.001	1.6	<0.01	**?**
Kunitz-type serine protease inhibitor 2, SPINT2	h	0.2	<0.001	0.3	<0.001	↓
aldehyde dehydrogenase 2, mitochondrial	c	0.3	<0.01	0.6	<0.01	↓
diaphorase (NADH), cytochrome b-5 reductase	c	0.5	<0.01		n.s.	**?**
superoxide dismutase 3, extracellular	c	0.4	<0.01	0.4	<0.001	↓
lipase A	c	2.6	<0.05		n.s.	**?**
protease inhibit, PI1, alpha-1-antitrypsin	h	5.5	<0.001			↑
GABA-B receptor 1A subunit	h	1.8	<0.001	n.e.		**?**
prostaglandin I2 prostacyclin synthetase	c	0.4	<0.05	0.81	<0,001	↓

**Gene Name**	**Platform**	**Atlas**		**Affymetrix**		**regulation**
**Functional group**	**Array type**	**ratio D/C**	***P *value**	**ratio D/C**	***P *value**	**ratio D/C**

**Protein turnover**						
purine-rich element-binding protein A	h	0.4	<0.01	0.6	<0.05	↓
glutathione S-transferase theta 1	h	0.4	<0.01	0.2	<0.05	↓
58-kDa inhibit. of RNA-activated protein kinase	h	0.3	<0.01			↓
G1/S-specific cyclin D1	h	0.5	<0.01		n.s.	**-**
carboxypeptidase H, CPE; enkephalin convertase	h	0.4	<0.01			↓
ribos. protein S6 kinase II α1, S6KII-alpha 1	h	3.1	<0.001		n.s.	**-**
carboxypeptidase N, polypeptide 1, 50 kD	c	2.0	<0.05	n.e.		**-**
eukariotic translation initiantion factor 4E bind prot 1		n.t.		2.9	<0.01	↑
60 S acdic ribosomal protein PO		n.t.		3.0	<0.01	↑
FOS-related antigen 1	h,c	4.9	<0.01	3.6	<0.05	↑
myc proto-oncogene	h	3.5	<0.01	2.8	<0.05	↑
ets domain protein elk-3; NET; SRF accessory protein	h	2.1	<0.001	4.4	<0.05	↑
adenine phosphoribosyltransferase		n.t.		2.1	<0.001	↑
high mobility group prot isoforms I & Y, HMGIY	h	10.1	<0.001	6,0	<0.01	↑
superoxide dismutase 2, mitochondrial	c	2.6	<0.01	3.7	<0.001	↑
**Cell receptors**						
Natriuretic peptide receptor A/guanylate cyclase A	c	0.4	<0.001	0.3	<0.001	↓
natriuretic peptide receptor C/guanylate cyclase C	c	0.5	<0.001		n.s.	**-**
protein tyrosine phosphatase receptor type F	c,h	0.4	<0.01	n.t.		**-**
**growth factors**						
vascular endothelial growth factor, VEGF	h	4.7	<0.05		n.s.	**-**
endothelin 2	c	0.6	<0.01	n.e.		**-**
fibroblast growth factor receptor1	h	0.6	<0.01	0.7	<0.05	↓
insulin-like growth factor-binding protein 2, IGFBP2	h	0.3	<0.001	0.2	<0.01	↓
cardiotrophin 1	c	0.4	<0.001	0.5	>0.05	↓
pleiotrophin, heparin-binding growth factor 8, HBGF8	h	0.5	<0.05	0.6	<0.05	↓
**inflammation + stress response**						
colony-stimulating factor 1 receptor, CSF1R	h	2.0	<0.05	1.7	<0.05	↑
myeloid cell nuclear differentiation antigen	h	3.5	<0.001	3.9	<0.05	↑
tumor necrosis fact recept superfam memb 1B	h	2.0	<0.001		n.s.	**-**
granulocyte colony stimul factor recept	h	3.5	<0.01	n.e.		**-**
interleukin 2 receptor alpha subunit, IL-2, CD25 antigen	h	3.1	<0.001	5.3	<0.001	↑
interleukin 6, IL-6	h	3.9	<0.01	4.3	<0.001	↑
interleukin 8, IL-8				12.2	<0.05	↑
small inducible cytokine subfamily A member 2	h,c	3.8	<0.01	2.2	<0.05	↑
90-kDa heat-shock proteinA HSP90A	h	2.6	<0.05	n.t.		↑
60-kDa heat shock protein HSP60	h	2.7	<0.05	n.t.		↑
CD9 antigen p24	c	0.5	<0.001	0.5	<0.01	↓
27-kDa heat shock protein HSP27	h	0.4	<0.001	n.t.		-
**blood, hemostasis**						
endothelial plasminogen activator inhibit 1, PAI1	h	2.8	<0.05	4.0	<0.05	↑
thrombomodulin	h	2.8	<0.05		n.s.	**-**
hexabrachion, tenascin C	c	2.6	<0.01	6.2	<0.05	↑
heme oxygenase 1	h	15.4	<0.05	3.6	<0.05	↑
tissue factor pathway inhibit, coagulation inhibit	c	2.0	<0.05	1.6	<0.05	↑
tissue factor pathway inhibitor 2	c	2.3	<0.05	3.0	<0.05	↑
**others**						
c-fgr proto-oncogene p55-FGR	h	2.6	<0.01	2.6	<0.01	↑
erbB3 proto-oncogene; HER3	h	3.7	<0.001	n.t.		↑
caspase 4	h	1.9	<0.05	n.t.		↑
follistatin-related protein	h	0.5	<0.01	0.6	<0.01	↓
Gem; induced immediate early protein	h	0.2	<0.001	0.3	<0.001	↓
parathymosin athymosin	h	0.6	<0.05	0.8	n.s.	**-**
prostaglandin I2 (prostacyclin) synthase	c	0.4	<0.05	n.t.	<0.001	↓
calcium-activated potassium channel beta subunit	h	0.3	<0.001	0.4	<0.01	↓
solute carrier fam 4 anion exchanger member 3	c	0.5	<0.001	n.t.		**-**

The direct comparison of differentially expressed genes according to platforms of Clontech Atlas and Affymetrix revealed a relatively high concordance between both data sets. Thus, of those genes which have been found differentially expressed on Clontech platform, 84% were also significantly differentially expressed on the Affymetrix platform. To illustrate this concordance, Affymetrix *p *values have been added to those genes reaching significance on the Clontech Atlas arrays (Table 2). Additionally, genes differentially expressed between dissected and control aorta were identified using the significance analysis of microarrays (SAM) software with 500 sample permutations [18].

#### b. Identification of genes of interest by SAM

Human and Cardiovascular cDNA arrays' (Clontech platform) data were used as normalized previously. Significant genes were selected independent of their expression levels when their score was above the specified threshold *delta*. With a FDR of 0 and by applying a minimal fold-change of 1.8 the analyses revealed consistent patterns of differential gene expression. In total, 37 genes were significantly differentially expressed, i.e., 23 genes were up- and 14 genes were down-regulated in dissected aorta samples as defined by Human cDNA array (Additional file 1). Additionally, 36 genes were differentially expressed, i.e., three genes were up- and 33 genes down-regulated in dissected aorta samples as defined by Cardiovascular array (Additional file 2). Applying SAM to Affymetrix data we firstly had to reduce the number of genes under analysis. Since the signal detection is much more precise for Affymetrix data than in case of Atlas filter arrays, we changed the cutoffs for the detectable differential gene expression and set the cutoff for minimal expression level to 30 RSU. 30 RSU is an absolute board line (TGT = 100) for signal detection on GeneChip, which we accept as measurable. We selected 11,128 expressed genes, and SAM has been used with a FDR of 0, and a minimal fold-change of 2.5 has been then applied. In total, 195 genes were significantly differentially expressed between dissected and control aorta. Of those, 60 were up-regulated and 135 were down-regulated (Additional file 3).

#### c. Partial least squares discriminant analysis (PLS-DA)

**Figure 1:**
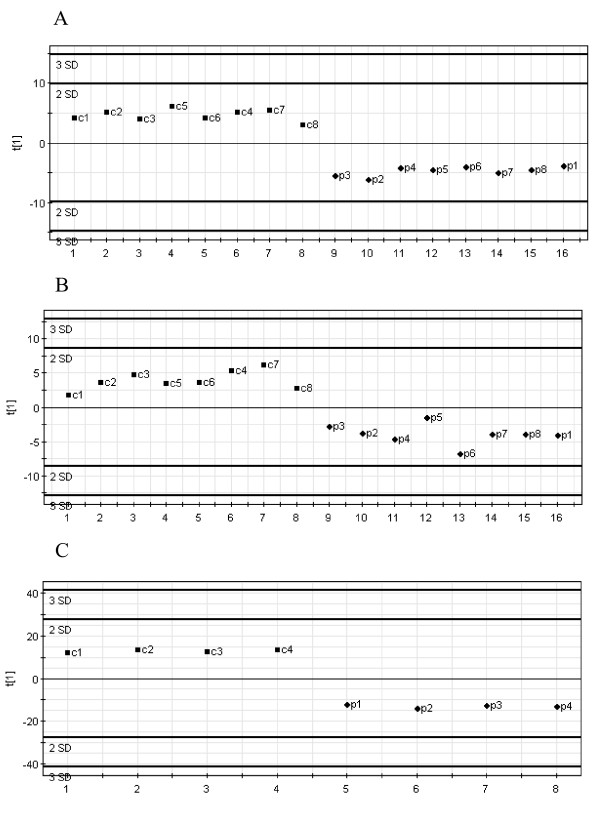
PLS-DA between control and dissected aorta samples. A, PLS discrimination with the 44 genes with *VIP *> 1.5 – Human Broad I array (Clontech). B, PLS discrimination with 30 genes with *VIP *> 1.32 – Cardiovascular array (Clontech). C, PLS discrimination with 79 genes with *VIP *> 1.51 – Affymetrix GeneChip microarray. *Horizontal axis*, number of samples in the table; *vertical axis*, the first PLS component. *Numbers next to the black squares *and *rhombus*, refer to control "c" or dissected "p" aorta samples.

We evaluated the discriminative ability of PLS-DA questioning for small gene sets to separate control and dissected aorta samples. Expression data obtained on the Human Atlas array were used in the PLS-DA analysis, which were carried out in the first iterative level with all 281 reliably expressed genes previously used in the *t *test. During the course of analysis, we selected those genes satisfying the cutoff criterion of having the variable importance in the projection (*VIP*) more than 1.5 with the first PLS component. In a second iterative PLS-DA only these genes (n = 44) were reanalyzed to avoid running an overfitted model (see genes listed in Additional file 4). The *VIP *values (Additional file 4) correspond to the model with selected 44 genes and display lower values than obtained in the model with all 281 genes. This phenomenon has been reported previously [18,19]. To demonstrate a good agreement between both statistical analyses, *t*-test and PLS-DA, we included *t*-test's *P *values alongside with in-between groups ratios into the Additional file 4. Results of PLS-DA analyzing the 44 selected genes are depicted in Fig. 1A. Apparently control and dissected aorta samples are clearly discriminated. We performed a permutation test for the model covering only 44 genes, which showed the robustness and precision of this model (Additional file 7).

Furthermore, we applied PLS-DA to the 149 reliably expressed genes, as detected on the Cardiovascular array (Clontech). After the analysis with one PLS component had been performed, we retained 30 genes with highest *VIP *values between 1.8 and 1.32 for the second iterative PLS analysis run. Fig. 1B shows PLS-DA scatter plot with one component perfectly subdividing control and dissected aorta samples. Selected genes are listed in Additional file 5. An independent permutation test showed that model with selected genes was the one with the highest predictive power (Additional file 7).

For the Affymetrix platform, PLS-DA was carried out on the original 11,128 probe sets that passed the QC filtering process. Although this process may lead to an over parameterized model with poor prediction properties, it provides a preliminary assessment of the most important discriminative variables. Further, those variables satisfying the criteria of minimal ratio of 2.5 were retained and with the highest *VIP *values ranging from the maximum of 1.56 to 1.51 in the first iterative PLS analysis (Additional file 6). From those, 79 genes were selected, which demonstrated the highest predictive power (Fig. 1C), as shown by permutation analysis (Additional file 7).

#### d. Comparison between three statistical methods

Since *t*-test may be problematic in multiple testing we applied "Significance analysis of microarrays" (SAM) and *"Partial least squares discriminant analysis" (PLS-DA) *to three data sets obtained by use of Human, Cardiovascular and Affymetrix GeneChip arrays. All three statistical methods were adequate in capturing a small number of the potentially relevant genes to the disease. We found the agreement between three statistical methods very high for all three array types. We have summarized the agreement between the three methods for each of used array type in the Table 3. Thus, we found 31 genes being differentially expressed between dissected and control aorta samples by applying the *t*-test to data obtained with Cardiovascular array. SAM analysis assorted 36 differentially expressed genes. Of those, 27 genes were on the *t*-test list. PLS-DA found 34 genes, which were sufficient to discriminate between both groups of samples. Of those, 26 genes were identified by the *t*-test.

**Table 3:** The level of agreement between the *t *test, SAM and PLS-DA for selecting differentially expressed genes from the same data set.

**Type of array**		**N.o. of differently expressed genes between dissected and control aorta**			**Agreement between all three methods**
		***t*-test *P *< 0.01**	**SAM**	**PLS-DA**	

**Human Broad I cDNA array**		**43**	**37**	**35**	**29**
	Agreement with *t*-test	-	29 (67%)	35 (81%)	-
**Cardiovascular cDNA array**		**31**	**36**	**34**	**22**
	Agreement with *t*-test	-	27 (87%)	26 (84%)	-
**Affymetrix HG-133A**		**242**	**195**	**79**	**79**
	Agreement with *t*-test	-	195 (81%)	79 (33%)	-

We performed the new *t*-test analysis for the Affymetrix data by setting another cutoff values. Additionally to the board line of 30 RSU for the detectable gene expression, minimal fold change was set to 2.5 for Affymetrix data and cut-off for *P*-value was < 0.01 for the *t*-test. Under this assumption 11,128 of 22,283 genes (50%) showed detectable expression on the Affymetrix platform, of which 242 genes (21.8%) were differentially expressed in dissected and control aorta with 89 genes showed higher and 153 – lower expression in dissected aorta. Of those, 195 genes were found by SAM. Consequently, 79 genes, which are the part of 195^th ^and 242^nd ^gene sets, were independently extracted by PLS-DA, as sufficient to discriminate between normal and diseased aorta samples. Therefore, expression profiling of dissected and control aorta revealed significant differences in expression levels of certain genes.

### Availability of raw expression data

Raw data for Clontech Human, Cardiovascular, and normalized expression data for Affymetrix HG-133A arrays have been submitted as Additional files 8, 9, 10 in Excel format.

### Validation of some genes differentially expressed in dissected and control aorta by TaqMan real-time PCR

We selected a subset of 24 genes that were influenced by aortic dissection process for confirmation using quantitative real-time PCR. All chosen genes encode for proteins, which are important for the aortic structure and function. Confirming the microarray data, we found that almost all genes except of hypoxia-inducible factor 1 (*HIF1*α) and metalloproteinase -11 (*MMP-11) *were up- or down-regulated in dissected samples (Table 4). Pearson correlation analysis revealed a significant correlation between the cDNA or oligonucleotide array data and real-time PCR data for expression changes. Notably, Affymetrix array and real-time PCR data showed on the average higher correlation than that obtained on the Atlas Clontech platform.

**Table 4:** Correlation of Clontech/Affymetrix gene expression levels with TaqMan real-time RT-PCR derived values. C means *Clontech array *(*BD Atlas™ Human1.2 Array *or *BD Atlas™ Human Cardiovascular Array*, A means *Affymetrix Human Genome U133A GeneChip*, MLCK * = myosin light chain kinase, MHCP 11** = myosin heavy chain polypeptide 11, KCNMB1*** = calcium-activated potassium channel beta, APEG1**** = Homo sapiens nuclear protein, HIF1α***** = hypoxia-inducible factor 1, alpha subunit.

Gene Name	Array	ABI Hs code	GenBank	Pearson correlation		Ratio (D/C) array	Ratio (D/C) RT-PCR
				*r*	*P*		
collagen VIII α1	C	00156669m1	X57527	0.626	8.36E-03	0.27	0.23
PKD1	C	00165489m1	U24497	0.497	4.78E-02	0.31	0.43
PKD2	C	00165517m1	U50928	0.655	5.11E-03	0.37	0.19
tenascin C	C	00233648m1	X78565	0.789	1.96E-04	2.56	5.68
elastin	C	00355783m1	M36860	0.676	3.42E-03	0.33	0.14
integrin α5	C	00233743m1	X06256	0.679	3.21E-03	3.5	1.93
decorin	C	00370385m1	M14219	0.715	1.48E-03	0.34	0.61
fibulin 5	A	00197064m1	AF112152.1	0.852	6.21E-07	0.47	0.16
MUC18	C	00174838m1	M28882	0.883	2.77E-06	0.31	0.18
MMP11	C	00171829m1	X57766	-0.036	0.89	2.1	0.57
TIMP2	C	00234278m1	J05593	0.521	3.63E-02	0.41	0.53
TIMP3	C	00165949m1	U14394	0.794	1.63E-04	0.2	0.47
vinculin	C	00243320m1	M33308	0.807	1.04E-04	0.22	0.31
actin α2	A	00426835g1	J05192.1	0.965	2.77E-12	0.52	0.19
actinin α4	A	00245168m1	U48734.1	0.946	1.03E-10	0.44	0.19
filamin A	C	00155065m1	X53416	0.874	4.91E-06	0.33	0.09
MLCK *	C	00178736m1	U48959	0.741	7.80E-04	0.36	0.19
MHCP 11**	A	00224622m1	NM0228	0.580	9.57E-03	0.48	0.09
leiomodin 1	A	00201704m1	BC001755.1	0.979	4,23E-14	0.43	0.19
heme oxygenase 1	C	00157965m1	X06985	0.837	3.09E-05	8.47	6.58
KCNMB1***	C	00188073m1	U25138	0.763	4.31E-04	0.29	0.29
calponin 1	A	00154543m1	U37019.1	0.985	2.76E-15	0.31	0.13
APEG1* ***	A	00195347m1	U57099.1	0.854	5.51E-07	0.58	0.19
HIF1α*****	A	00153153m1	U22431.1	-0.932	0.27	1.7	1.86

### Gene groups with predominant down-regulation in dissected aorta

Differentially expressed genes could be subdivided into several functional groups (Table 2).

#### Extracellular matrix (ECM)

Several genes that encode for extracellular matrix proteins displayed lower expression in dissected aortic wall relative to normal aortic wall (Table 2). Collagen distribution on the Atlas Clontech arrays: Whereas the mRNA of collagen 1, -IV α1, -XV, -XVI were equally distributed in dissected and control aorta, collagen II, -III, -IV α*6*, -V, -IX, -XVIII, -XIX were not expressed on the cardiovascular membrane. However, genes for collagen VI α3, -VIII α1, -VIII α2, – XIV showed a lower gene expression in dissected compared to control aorta. Collagen distribution on the Affymetrix chip: The Affymetrix chip displayed similar collagen gene expressions compared to the Atlas experiment. Genes for collagen IV α2, -IV α5, -VIII α1, -VIII α2, -XV, -XVIII α1 displayed a lower gene expression in dissection, whereas RNAs for collagen I, -III, -IV α1, -V and -VI were equally distributed, while the other collagens did not show any significant levels of gene expression. The lower gene expression of collagen VIII was also confirmed by RT-PCR. The mRNA of gene for elastin, an important connective tissue fiber, was reduced in dissected aorta, which was validated by RT-PCR.

#### Cytoskeleton and myofibrillar genes

Another group of genes with decreased expression levels consists of cytoskeleton and myofibrillar genes. We found that expression of α-actin gene, actinin α2 and -α4, tropomyosin, gelsolin and some myosin light and heavy chains were down-regulated in dissected aorta compared to normal control aorta.

#### Cell-cell adhesion

Expression levels of genes, such as gene for fibulin 5 and integrin α7B, cell surface glycoprotein *MUC18 *and polycystin 1 and -2 coding for cell-cell adhesion proteins were significantly lower in the dissected aorta.

#### Matrix metalloproteinases and their inhibitors

Despite the connective tissue components had shown similarity in their expression levels and degree of differential gene expression on both array platforms, the expression of the matrix metalloproteinases *(MMP) *and their inhibitors was less uniform. On the Affymetrix platform only *MMP-14 *and *MMP-19 *were up-regulated, whereas *MMP-2 *and *MMP-9 *were equally expressed in dissected and control aorta. Genes of other metalloproteinases were not expressed. *TIMP1*, *TIMP2*, and *TIMP3 *were consistently expressed in both, dissection and control. In contrast, expression of *MMP-11 *and *TIMP1 *was up-regulated in dissection, as found on Clontech platform, whereas *TIMP2 *and *TIMP3 *were down-regulated. The differential expression of both, *TIMP2 *and *TIMP3 *was confirmed by real time PCR.

### Gene groups with predominant up-regulation in dissected aorta

#### Inflammation and apoptosis

Expression of several genes associated with inflammation processes, e.g. genes coding for the inflammatory chemokines, as interleukines *(IL*), *IL-2*, *IL-6 *and *IL-8*, were up-regulated in dissected aorta. Some stress response genes, as 90-kDa heat-shock protein A (*HSP90A*), *HSP60*, and *HSP27 *were also up-regulated in dissection. Only one gene related to apoptosis, caspase 1 was up-regulated in dissection.

#### Blood and hemostasis

Genes of some blood components and components of coagulation and lysis pathways, for example, gene for heme oxygenase 1, endothelial plasminogen activator inhibitor, *PAI1 *or thrombomodulin were up-regulated in the freshly dissected aorta.

#### Proliferation, transcription and translation

We observed differential expression of some genes related to cell growth in dissection and normal aorta, some genes were up-regulated, others down-regulated.

## Discussion

Molecular features underlying the pathogenesis of aortic dissection are still enigmatic. Knowledge of the differential gene expression in dissected compared to the normal aorta may help to identify candidate genes for further studies allowing insights into the pathogenesis of aortic dissection. We proposed that some stable disfunctions in gene expression may underlie the development of aortic dissection. Sixty-six differentially expressed genes have been found and described in our previous study of aortic dissection [12]. Up-regulated genes in aortic dissection were involved in inflammation, extracellular matrix proteolysis, proliferation, transcription and translation processes. Down-regulated genes coded for extracellular matrix, adhesion and cytoskeletal proteins. Although the differences were significant, insufficient number of genes on the cDNA array and the absence of any verification of differential gene expression made the results of preliminary character.

In the present study we analyzed the gene expression in the aortic wall of acute Stanford A dissection and compared it with normal control aorta using two different microarray platforms followed by independent verification by real-time RT-PCR. We followed different strategies to study aortic dissection further and verify our previous and new findings concerning differentially expressed genes. First of all, we collected new tissue samples. Whereas multiorgan donors in our pilot study were significantly younger than patients with aortic dissection disease, we brought the age of both groups into line. The samples were analyzed with the previously used cDNA array (Atlas Human Broad I). Additionally, we used the Atlas Human Cardiovascular array containing probes for genes, which are highly specific for cardiovascular systems. Since some genes were in common on both Clontech filter arrays, an internal control was possible to verify the results from the Clontech platform. As a further result, we found the same genes being up- or down-regulated compared with our first study, thus confirming the findings from our pilot study [12]. Although data on expression profiles of control and dissected aorta has been confirmed between groups and arrays used in both studies, pilot and the present one, the further extension of genes under analysis was definite necessarily.

Eight samples, four aortic samples obtained from patients with type A acute aortic dissection and four control samples obtained from multiorgan donors, were studied on Affymetrix microarrays with more than 22,000 features corresponding human genes/ESTs. About 60% of the genes with altered expression in the dissected versus normal aorta were down-regulated in the dissected aorta. An interesting observation is the high percentage of down-regulated genes that are related to extracellular matrix, cell to cell adhesion, and cytoskeleton structures. It suggests that pathology of aortic dissection results from a loss of gene function. Smooth muscle cells (SMCs) in the media where the dissection occurs either seem to produce less proteins maintaining the stability of the aortic wall structure, or the number of SMCs themselves expressing these genes is reduced [23,24].

### Extracellular matrix (ECM)

The human aorta consists of three distinct layers: tunica intima, tunica media, and tunica adventitia. The media where the dissecting process takes place is the thickest layer in cross section and contains fenestrated elastin separated by smooth muscle cells, collagen chains and ground substance such as proteoglycans and glycoproteins [25-27]. Human aorta contains 12–24 g collagen and 28–32 g elastin per 100 g tissue [26]. Whereas collagen fibers provide strength and resistance to stretching, elastin provides elasticity and is very essential in the ascending aorta especially close to the heart [24-29]. The major types of collagen found in dissected and control aorta are collagen I and III [30-32]. Type IV collagen, the major component of basement membranes and basal laminae surrounding smooth muscles and nerves [26,29,30], is a family of six homologous chains (α1–α6) that have tissue-specific distribution. Heavily thickened type IV collagen structures surrounding individual smooth muscle cells were found in fibrous plaques but never in unaffected intima [30,31]. The chains of type IV collagen are assembled into supramolecular networks that differ in the chain composition. Six chains of type IV collagen are distributed in three major networks α1/α2, α3/α4/α5, and α1/α2/α5/α6 [32].

Gene expression of collagen IV α2 and -α5 was down-regulated in the dissected aorta. Sariola [33] had already shown defects in type IV collagen around medial smooth muscle cells when analyzing dissected aorta by immunohistochemistry. Defects in smooth muscle cell basal laminae were found throughout the media in cystic medial degeneration and in medionecrosis. Similar to our experiment no defects in the expression of interstitial collagens type I and III were seen in the dissecting aortas. The authors concluded that local changes in the basement laminae of the medial layer are important in the pathogenesis of the aortic dissection. This observation was confirmed by Ishii and Asuwa who also compared dissected and normal aortic wall by immunohistochemistry [34]. They also detected a significant higher concentration of MMP-2 and MMP-9 proteins at the beginning of dissection near the „entry" whereas in intact areas of the same aorta MMP-2 and MMP-9 proteins were not increased. We did not observe any differential expression of *MMP-2 *and *MMP-9 *in dissected and control aorta. A possible explanation is that our gene expression studies considered the whole aortic wall and not especially the „entry site" of the dissection.

Apart from collagen IV we found a decreased gene expression of collagen XVIII and of the proteoglycan decorin in the dissected aorta, which proteins have a high affinity to collagen I and III fibrils.

Studies of aortic aneurysms have already shown a significant reduction of elastin content in the diseased aortic wall, which probably occurs due to a high elastase activity [35,36]. We were also able to demonstrate a reduced gene expression of elastin in dissection nevertheless in contrast to aneurysmal degradation, we could not detect elastase (*MMP-12*) expression on the array. On the contrary, our Affymetrix experiments showed a higher gene expression of the matrix metalloproteinases 11,-14 and -19. MMP-11 and MMP-14 degrade fibronectin and proteoglycanes, and MMP-14, additionally, – the major collagens I-III [37]. MMP-19, a recently discovered member of the zinc-dependent proteolytic enzymes, is able to process various proteins of the basement membranes [38,39]. Titz and colleagues treated tumor extracellular matrix with recombinant MMP-19 and analyzed its effect on capillary like formation [39]. MMP-19 preferentially cleaves nidogen-1, which interferes with its cross link abilities for extracellular matrix proteins, especially laminin-1 and type IV collagen. As a consequence, the capillary-like structure formation was inhibited. The authors concluded that MMP-19 might be one of the enzymes that interferes with stabilization or maturation of nascent vasculature. We believe that MMP-19 might play a key role in propagating dissection by degrading type IV collagen in the vasa vasorum and the basal laminae surrounding smooth muscle cells in the media.

The ECM contains many glycoproteins which bind to cell surface components, and mediate cell-matrix connections and cell to cell contacts [26]. We detected decreased expression levels of some of these genes in the dissected aorta, for example, genes coding for fibulin 5, cell surface glycoprotein MUC18, SPARC-like 1 (osteonectin), clusterin and connexin 43. Additionally, expression of several integrin genes was also decreased in diseased aorta. Integrins are adhesion receptors on the cell surface. Tissue remodeling is dependent on integrin-matrix signaling and interaction [27].

In our pilot study [12] we described that expression of polycystin 1 was lower in dissected aorta samples. In the present study we observed decreased expression of both genes for polycystin 1 and -2. Polycystins function as matrix receptors to link ECM to the actin cytoskeleton via focal adhesion proteins [40,41]. Mutations of these genes cause autosomal dominant polycystic kidney disease and familiar clustering of aortic dissection events, and supraaortal dissection or cerebral artery aneurysm in this disease have already been described [42].

### Smooth muscle and cytoskeleton proteins

We noticed reduced expression levels of some myofibrillar genes belonging to the contractile system of smooth muscle cells in dissection. A possible explanation could be a depletion of smooth muscle cells in the media of the dissected aorta. However, loss of contractility means reduced strength in resisting to pulsating hemodynamic forces.

The cytoskeleton is a dynamic intracellular network influencing cell morphology and motility as well as intracellular transport. The cytoskeleton is composed of three principal types of protein filaments: actin filaments, intermediate filaments and microtubuli. The major cytoskeletal protein in most cells is actin, which polymerizes to form actin filaments (microfilaments). These filaments are organized in actin bundles. Actin binds a lot of cross-linking proteins [27]. In our studies gene expression of actin α2 itself and some of the actin-binding proteins (actinin α1 and -α4, vinculin, gelsolin, filamin A, macrophin or actin binding protein ABP620) were reduced in dissection.

### Inflammation

A lot of genes being involved in inflammation were up-regulated in the dissected aorta, *e.g*., the inflammatory chemokines *IL-2*, *IL-6 *and *IL-8*, or integrin αM and integrin α2, which bind to lymphocytes or macrophages.

### Protein turnover

Our recent pilot study had shown an upregulation of several genes being involved in cell proliferation and considered this as result of an injury and repair mechanism [12]. In our new comparative gene expression studies some transcription and translation factors were up-regulated, whereas others were down-regulated.

### Blood and hemostasis

Some genes being substantially important for blood cells as heme oxygenase 1 or for blood coagulation and lysis such as endothelial plasminogen activator inhibitor 1 were up-regulated in dissection. This may be a consequence of the dissection process itself. In our recent study we have compared nondissected and dissected aortic wall areas of the same tissue sample and had found no significant difference in their gene expression patterns [12]. Nevertheless, Stanford A patients undergo emergency surgery within 24 h after acute dissection and there may be some blood components left in the tunica media.

## Conclusion

Although the clinical significance of the present study cannot be definitely estimated yet, the data on differential gene expression illustrate the underlying biological processes involved in aortic dissection formation. Gene expression studies comparing acutely dissected ascending aorta and normal control aorta have shown major differences. Whereas some genes mediating inflammation were up-regulated in dissection, a lot of genes mediating stability and integrity of the aortic wall were down-regulated. Aortic dissection seems to be the dramatic endpoint of a longer lasting process of degeneration and insufficient remodeling of the aortic wall. Expression profiling of aortic dissection was capable of detecting biologically important changes in gene expression, which are probably associated exclusively with aortic dissection with excellent fidelity while revealing important down-stream effects of tissue remodeling and dysfunction of aortic structures for future in-depth studies. Indeed, we already examined the expression and localization of some extracellular matrix components, cytoskeleton and adhesion proteins in a further immunohistochemical study of normal and dissected aorta (manuscript in preparation).

## Competing interests

The author(s) declare that they have no competing interests.

## Authors' contributions

B. T. Weis-Müller and O. Modlich evenly contributed to this study and share the first-authorship. Experiments and data analysis were mainly done by Dr. O. Modlich. Dr. B.T. Weis-Müller covered the clinical part of the study and wrote the paper.

## Supplementary Material

Additional File 1 *Genes identified by SAM as differentially expressed between dissected and control groups by use of Human Clontech cDNA arrays*. Click here for file

Additional File 2 *Genes identified by SAM as differentially expressed between dissected and control groups by use of Cardiovascular Clontech cDNA arrays*. Click here for file

Additional File 3 *Genes identified by SAM as differentially expressed between dissected and control groups by use of Affymetirx HG-U133A microarrays*. Click here for file

Additional File 4 *Genes involved in discrimination between control and dissected aorta samples ordered by VIP as defined by SIMCA-P software using Human arrays (Clontech platform). PLS-DA results are listed together with P values from t test for corresponding genes for Human and Affymetrix arrays*. Click here for file

Additional File 7 Validation of PLS-DA for control *versus *dissected aorta samples by permutation. A, by use of 44 genes selected from Human Broad I array, Clontech platform. B, by use of 30 genes selected from Cardiovascular cDNA array, Clontech platform. C, by use of 79 gene probes selected from the Affymetrix GeneChip microarray. The *horizontal axis *is the value *R*^2 ^and *Q*^2^. The two values on the right hand corner *r *= 1 correspond to the values of value *R*^2 ^and *Q*^2 ^for the actual data. Each *symbol *represents a result of permutation. Click here for file

Additional File 5 *Genes involved in discrimination between control and dissected aorta samples ordered by VIP as defined by SIMCA-P software using Cardiovascular arrays (Clontech platform). PLS-DA results are listed together with P values from t test for corresponding genes for Cardiovascular and Affymetrix arrays*. Click here for file

Additional File 6 *Genes involved in discrimination between control and dissected aorta samples ordered by VIP as defined by SIMCA-P software using data from Affymetrix platform. PLS-DA results are listed together with P values from t test for corresponding genes for Affymetrix arrays*. Click here for file

Additional File 8 *Contains scaled to fixed target signal intensity TGT of 100 RSU fluorescent intensities of all hybridization probes obtained from HG-133A microarrays (Affymetrix platform)*. Click here for file

Additional File 9 *Contains raw expression data obtained from Human Broad I arrays (Clontech platform)*. Click here for file

Additional File 10 *Contains raw expression data obtained from Cardiovascular arrays (Clontech platform)*. Click here for file
