# Supplementary material for: Gene expression in acute Stanford type A dissection: a comparative microarray study
Source: J Transl Med. 2006 Jul 6;4:29. doi: 10.1186/1479-5876-4-29 (PMC1557406; doi:10.1186/1479-5876-4-29)
Supplement: Additional File 1 — Genes identified by SAM as differentially expressed between dissected and control groups by use of Human Clontech cDNA arrays. [file 1479-5876-4-29-S1.doc]

**Supplemental Table 1. Genes identified by SAM as differentially expressed between dissected and control groups by use of Human Clontech cDNA arrays.**

| Gene ID | Gene Name | Score | Numerator(r) | Denominator | Fold change | q- avalue ( (%) | | Local  FDR(%) | |
| --- | --- | --- | --- | --- | --- | --- | --- | --- | --- |
| E08f | high mobility group protein isoforms I & Y (HMGIY) | 7.19 | 6.72 | 0.93 | 10.06 | 0 | 0.224 | |  |
| E08i | fibronectin receptor alpha subunit (FNRA); integrin alpha 5 | 6.24 | 0.56 | 0.09 | 3.50 | 0 | 0.000 | |  |
| B03l | ribosomal protein S6 kinase II α 1 | 5.59 | 1.01 | 0.18 | 3.14 | 0 | 0.000 | |  |
| D05d | GABA-B receptor 1A subunit | 5.40 | 0.47 | 0.09 | 1.80 | 0 | 0.000 | |  |
| A11l | myeloid cell nuclear differentiation antigen (MNDA) | 5.19 | 1.47 | 0.28 | 3.51 | 0 | 0.000 | |  |
| D10j | ets domain protein elk-3; (SAP2) | 4.97 | 2.48 | 0.50 | 2.08 | 0 | 0.000 | |  |
| F05k | protease inhibitor 1 (PI1) | 4.93 | 3.80 | 0.77 | 5.54 | 0 | 0.000 | |  |
| B12l | phospholipase C beta 2 (PLCB2) | 4.69 | 0.12 | 0.03 | 3.44 | 0 | 0.061 | |  |
| A09l | 120-kDa nucleolar protein 1 (NOL1) | 4.61 | 0.38 | 0.08 | 2.58 | 0 | 0.290 | |  |
| C09b | bone marrow stromal antigen 1; CD157 | 4.53 | 0.23 | 0.05 | 2.28 | 0 | 0.546 | |  |
| A11g | matrix metalloproteinase 11 (MMP11) | 4.51 | 7.57 | 1.68 | 2.12 | 0 | 0.605 | |  |
| E01l | IL2 receptor alpha; CD25 antigen | 4.46 | 10.97 | 2.46 | 3.10 | 0 | 0.783 | |  |
| C14l | activator 1 37-kDa subunit; RFC4 | 4.31 | 0.34 | 0.08 | 2.39 | 0 | 1.467 | |  |
| C09j | IEX-1L anti-death protein; PRG-1 | 4.27 | 0.39 | 0.09 | 2.58 | 0 | 1.663 | |  |
| A12e | erbB3 proto-oncogene; HER3 | 4.24 | 4.75 | 1.12 | 3.66 | 0 | 1.810 | |  |
| F13i | interleukin 6 (IL6) | 4.11 | 4.39 | 1.07 | 3.92 | 0 | 2.666 | |  |
| A09m | membrane protein E16 (MPE16) | 4.01 | 0.19 | 0.05 | 3.46 | 0 | 3.348 | |  |
| F08g | leukemia inhibitory factor (LIF) | 3.98 | 0.19 | 0.05 | 5.03 | 0 | 3.633 | |  |
| A12c | myc proto-oncogene | 3.95 | 18.74 | 4.75 | 3.49 | 0 | 3.902 | |  |
| B13d | CXC chemokine receptor type 4 (CXCR4); fusin | 3.91 | 0.50 | 0.13 | 3.32 | 0 | 4.225 | |  |
| B02m | phosphatidylinositol 3-kinase catalytic subunit delta isoform | 3.87 | 0.14 | 0.04 | 2.94 | 0 | 4.613 | |  |
| F05n | tissue inhibitor of metalloproteinase 1 (TIMP1) | 3.81 | 7.12 | 1.87 | 1.83 | 0 | 5.238 | |  |
| D01m | E2F transcription factor 3 (E2F3) | 3.63 | 0.14 | 0.04 | 1.93 | 0 | 7.353 | |  |
| A01n | calcium-activated potassium channel beta subunit (KCNMB1) | -10.57 | -0.71 | 0.07 | 0.29 | 0 | 1.063 | |  |
| A08g | cell surface glycoprotein MUC18 | -6.49 | -6.42 | 0.99 | 0.31 | 0 | 1.852 | |  |
| F06n | tissue inhibitor of metalloproteinase 2 (TIMP2) | -5.84 | -3.52 | 0.60 | 0.48 | 0 | 1.807 | |  |
| F14l | Kunitz-type serine protease inhibitor 2; placental bikunin | -5.81 | -3.30 | 0.57 | 0.23 | 0 | 1.807 | |  |
| B12g | smooth muscle & non-muscle myosin light chain kinase (MLCK) | -5.58 | -4.71 | 0.84 | 0.36 | 0 | 1.816 | |  |
| B06m | Gem; induced immediate early protein | -5.37 | -4.68 | 0.87 | 0.24 | 0 | 1.845 | |  |
| A09g | IGF-binding protein 2 | -5.23 | -7.13 | 1.36 | 0.27 | 0 | 1.887 | |  |
| E02a | nuclear factor I-X (NFI-X) | -5.22 | -0.25 | 0.05 | 0.54 | 0 | 1.891 | |  |
| F05b | 27-kDa heat shock protein (HSP27) | -5.10 | -152.9 | 30.00 | 0.42 | 0 | 1.950 | |  |
| E13i | integrin alpha 7B (IGA7B) | -5.06 | -0.83 | 0.16 | 0.37 | 0 | 1.973 | |  |
| E06j | polycystin (PKD1) | -4.73 | -5.71 | 1.21 | 0.31 | 0 | 2.385 | |  |
| F06k | carboxypeptidase H (CPH) | -4.13 | -2.62 | 0.63 | 0.44 | 0 | 4.689 | |  |
| C13j | clusterin (CLU) | -3.87 | -5.91 | 1.52 | 0.47 | 0 | 6.683 | |  |
| C11d | 58-kDa inhibitor of the RNA-activated protein kinase | -3.86 | -1.36 | 0.35 | 0.33 | 0 | 6.810 | |  |
