# Supplementary material for: Gene expression in acute Stanford type A dissection: a comparative microarray study
Source: J Transl Med. 2006 Jul 6;4:29. doi: 10.1186/1479-5876-4-29 (PMC1557406; doi:10.1186/1479-5876-4-29)
Supplement: Additional File 2 — Genes identified by SAM as differentially expressed between dissected and control groups by use of Cardiovascular Clontech cDNA arrays. [file 1479-5876-4-29-S2.doc]

**Supplemental Table 2. Genes identified by SAM as differentially expressed between dissected and control groups by use of Cardiovascular Clontech cDNA arrays.**

| Gene ID | Gene Name | | Score | | Numerator | | Denomi-  nator | | Fold Change | | q-value (%) | | Local  FDR (%) | |
| --- | --- | --- | --- | --- | --- | --- | --- | --- | --- | --- | --- | --- | --- | --- |
| B3i | aquaporin 2 | 5.31 | | 0.39 | | 0.07 | | 2.69 | | 0 | | 8.137 | |  |
| F7j | GRO1 oncogene | 5.02 | | 0.44 | | 0.09 | | 5.43 | | 0 | | 8.575 | |  |
| D3m | tissue inhibitor of metalloproteinase 1 (TIMP1) | 4.11 | | 40.00 | | 9.74 | | 1.88 | | 0 | | 8.743 | |  |
| A3m | low-density lipoprotein receptor (LDLR) | -6.89 | | -7.40 | | 1.07 | | 0.37 | | 0 | | 0.067 | |  |
| A6c | natriuretic peptide receptor A/guanylate cyclase A | -6.80 | | -3.68 | | 0.54 | | 0.42 | | 0 | | 0.060 | |  |
| D1n | cardiotrophin 1 | -6.05 | | -2.18 | | 0.36 | | 0.42 | | 0 | | 0.000 | |  |
| D1c | adducin 1 (alpha) | -6.04 | | -0.33 | | 0.05 | | 0.37 | | 0 | | 0.000 | |  |
| C2n | filamin A, alpha (actin binding protein 280) | -5.93 | | -31.89 | | 5.38 | | 0.35 | | 0 | | 0.000 | |  |
| D3n | tissue inhibitor of metalloproteinase 2 (TIMP2) | -5.17 | | -5.37 | | 1.04 | | 0.44 | | 0 | | 0.000 | |  |
| A6e | natriuretic peptide receptor C/guanylate cyclase C | -4.98 | | -1.68 | | 0.34 | | 0.50 | | 0 | | 0.000 | |  |
| C3d | SPARC-like 1 (mast9, hevin) | -4.78 | | -23.28 | | 4.88 | | 0.35 | | 0 | | 0.000 | |  |
| A3b | connective tissue growth factor | -4.73 | | -0.32 | | 0.07 | | 0.16 | | 0 | | 0.000 | |  |
| F3m | collagen, type XIV, alpha 1 (undulin) | -4.55 | | -2.91 | | 0.64 | | 0.39 | | 0 | | 0.000 | |  |
| F7m | melanoma cell adhesion molecule | -4.37 | | -0.88 | | 0.20 | | 0.31 | | 0 | | 0.000 | |  |
| D1i | vinculin | -4.30 | | -0.89 | | 0.21 | | 0.19 | | 0 | | 0.000 | |  |
| B3l | solute carrier family 4, member 3 | -4.24 | | -1.46 | | 0.35 | | 0.51 | | 0 | | 0.000 | |  |
| B5n | CD9 antigen (p24) | -4.14 | | -2.31 | | 0.56 | | 0.53 | | 0 | | 0.000 | |  |
| D4a | tissue inhibitor of metalloproteinase 3 (TIMP3) | -4.14 | | -1.17 | | 0.28 | | 0.25 | | 0 | | 0.000 | |  |
| F4h | annexin A3 | -4.02 | | -0.76 | | 0.19 | | 0.16 | | 0 | | 0.125 | |  |
| D7n | mevalonate (diphospho) decarboxylase | -3.96 | | -0.16 | | 0.04 | | 0.51 | | 0 | | 0.337 | |  |
| D4e | sterol regulatory element binding transcription factor 2 | -3.89 | | -0.38 | | 0.10 | | 0.50 | | 0 | | 0.623 | |  |
| E5c | aldehyde dehydrogenase 2 family (mitochondrial) | -3.79 | | -0.71 | | 0.19 | | 0.34 | | 0 | | 1.069 | |  |
| A5d | vasoactive intestinal peptide receptor 2 | -3.60 | | -0.25 | | 0.07 | | 0.13 | | 0 | | 2.321 | |  |
| C6l | collagen, type VIII, alpha 2 | -3.55 | | -0.10 | | 0.03 | | 0.09 | | 0 | | 2.692 | |  |
| C3l | integrin, alpha 8 | -3.52 | | -0.46 | | 0.13 | | 0.07 | | 0 | | 2.987 | |  |
| E5a | aldehyde dehydrogenase 1 family, member A1 | -3.35 | | -0.19 | | 0.06 | | 0.19 | | 0 | | 4.719 | |  |
| B5e | superoxide dismutase 3, extracellular | -3.34 | | -11.30 | | 3.39 | | 0.46 | | 0 | | 4.885 | |  |
| F1h | galactosidase, alpha | -3.33 | | -0.38 | | 0.11 | | 0.38 | | 0 | | 4.964 | |  |
| A4i | phospholipase A2 receptor 1, 180kD | -3.33 | | -0.31 | | 0.09 | | 0.34 | | 0 | | 4.990 | |  |
| D7f | 3-oxoacid CoA transferase | -3.25 | | -0.41 | | 0.13 | | 0.35 | | 0 | | 6.043 | |  |
| A2d | endothelin 2 | -3.19 | | -0.84 | | 0.26 | | 0.54 | | 0 | | 6.792 | |  |
| A7g | protein tyrosine phosphatase, receptor type, F | -3.15 | | -0.73 | | 0.23 | | 0.53 | | 0 | | 7.468 | |  |
| A2l | vascular endothelial growth factor B | -3.07 | | -0.37 | | 0.12 | | 0.45 | | 0 | | 8.744 | |  |
| E4l | alcohol dehydrogenase 5 (class III) | -3.07 | | -0.47 | | 0.15 | | 0.35 | | 0 | | 8.751 | |  |
| C6k | collagen, type VIII, alpha 1 | -3.02 | | -1.03 | | 0.34 | | 0.24 | | 0 | | 9.480 | |  |
| A7d | leukotriene b4 receptor | -3.00 | | -0.30 | | 0.10 | | 0.55 | | 0 | | 9.886 | |  |
