# Supplementary material for: Gene expression in acute Stanford type A dissection: a comparative microarray study
Source: J Transl Med. 2006 Jul 6;4:29. doi: 10.1186/1479-5876-4-29 (PMC1557406; doi:10.1186/1479-5876-4-29)
Supplement: Additional File 4 — Genes involved in discrimination between control and dissected aorta samples ordered by VIP as defined by SIMCA-P software using Human arrays (Clontech platform). PLS-DA results are listed together with P values from t test for corresponding genes for Human and Affymetrix arrays. [file 1479-5876-4-29-S4.doc]

**Supplemental Table 4. Genes involved in discrimination between control and dissected aorta samples ordered by *VIP* as defined by SIMCA-P software using Human arrays (Clontech platform). PLS-DA results are listed together with *P* values from *t* test for corresponding genes for Human and Affymetrix arrays.**

| Gene Name | GenBank | *VIP* | *P* value; *t*-test | ratio (D/C) | *P* value;  Affymetrix | ratio (D/C);  Affymetrix |
| --- | --- | --- | --- | --- | --- | --- |
| calcium-activated potassium channel beta subunit  (KCNMB1) | U25138 | 1.22 | 4.27E-08 | 0.29 | 1.39E-03 | 0.37 |
| high mobility group protein isoforms I & Y (HMGIY) | M23619 | 1.15 | 4.59E-06 | 10.06 | 1.73E-03 | 6.02 |
| cell surface glycoprotein MUC18 | M28882 | 1.12 | 1.42E-05 | 0.31 | 2.02E-04 | 0.47 |
| fibronectin receptor alpha subunit; integrin alpha 5 | X06256 | 1.11 | 2.02E-05 | 3.50 | 3.47E-03 | 3.38 |
| tissue inhibitor of metalloproteinase 2 (TIMP2) | J05593 | 1.09 | 4.28E-05 | 0.48 | 8.63E-02 | 0.76 |
| Kunitz-type serine protease inhibitor 2; placental bikunin | U78095 | 1.09 | 4.45E-05 | 0.23 | 4.14E-05 | 0.3 |
| ribosomal protein S6 kinase II α 1 | L07597 | 1.08 | 6.43E-05 | 3.14 | 3.48E-03 | 2.86 |
| smooth muscle & non-muscle myosin light chain kinase (MLCK) | U48959 | 1.08 | 6.69E-05 | 0.36 | 1.8E-04 | 0.45 |
| GABA-B receptor 1A subunit | Y11044 | 1.07 | 8.79E-05 | 1.80 | Abs.^1^ | - |
| Gem; induced immediate early protein; ras family member | U10550 | 1.06 | 9.82E-05 | 0.24 | 2.17E-04 | 0.34 |
| SWI/SNF-related matrix-associated actin-dependent regulator of chromatin subfamily A member 2 | D26155 | 1.06 | 1.18E-04 | 0.61 | 4.53E-04 | 0.66 |
| insulin-like growth factor-binding protein 2 | M35410 | 1.06 | 1.28E-04 | 0.27 | 2.85E-03 | 0.24 |
| myeloid cell nuclear differentiation antigen (MNDA) | M81750 | 1.05 | 1.34E-04 | 3.51 | 1.27E-02 | 3.94 |
| 27-kDa heat shock protein (HSP27) | X54079 | 1.05 | 1.62E-04 | 0.42 | 5.54E-04 | 0.52 |
| integrin alpha 7B (IGA7B) | X74295 | 1.04 | 1.68E-04 | 0.37 | 5.99E-04 | 0.27 |
| ets domain protein elk-3 (SAP2) | Z36715 | 1.04 | 2.05E-04 | 2.08 | No exp.^2^ | - |
| protease inhibitor 1 (PI1) | X02920 | 1.03 | 2.20E-04 | 5.54 | 6.58E-03 | 6.09 |
| alpha1 catenin (CTNNA1) | D13866 | 1.02 | 3.00E-04 | 0.64 | 0.07 | 0.9 |
| Polycystin (PKD1) | U24497 | 1.02 | 3.21E-04 | 0.31 | 1.06E-03 | 0.36 |
| 120-kDa nucleolar protein 1 | X55504 | 1.01 | 3.85E-04 | 2.58 | 5.77E-03 | 1.5 |
| matrix metalloproteinase 11 (MMP11); stromelysin 3 | X57766 | 0.99 | 4.88E-04 | 2.12 | No exp. | - |
| IL2 receptor alpha; CD25 | X01057 | 0.99 | 5.35E-04 | 3.10 | No exp. | - |
| IEX-1L anti-death protein; PRG-1 | AF071596 | 0.98 | 7.39E-04 | 2.58 | Abs. | - |
| erbB3 proto-oncogene; HER3 | M29366 | 0.97 | 8.14E-04 | 3.66 | No exp. | - |
| carboxypeptidase H (CPH) | X51405 | 0.96 | 1.01E-03 | 0.44 | Abs. | - |
| solute carrier family 9 member 1 | M81768 | 0.96 | 1.01E-03 | 1.38 | 0.064 | 1.55 |
| interleukin 6 (IL6) | X04602 | 0.96 | 1.06E-03 | 3.92 | 0.02 | 4.3 |
| guanine nucleotide-binding protein α stimulating activity polypeptide 1 | M14631 | 0.95 | 1.35E-03 | 0.69 | 2.12E-04 | 0.71 |
| urokinase-type plasminogen activator receptor GPI-anchored form (U-PAR) | U08839 | 0.94 | 1.38E-03 | 1.53 | 3.91E-03 | 2.61 |
| myc proto-oncogene | V00568 | 0.94 | 1.46E-03 | 3.49 | 2.02E-02 | 2.73 |
| neurotrophin 3 (NT3) | X53655 | 0.94 | 1.47E-03 | 0.71 | 8.96E-04 | 0.49 |
| CXC chemokine receptor type 4 | D10924 | 0.94 | 1.52E-03 | 3.32 | 1.56E-02 | 2.7 |
| clusterin (CLU) | M74816 | 0.93 | 1.68E-03 | 0.47 | 1.09E-02 | 0.48 |
| 58-kDa inhibitor of the RNA-activated protein kinase | U28424 | 0.93 | 1.72E-03 | 0.33 | No exp. | - |
| tissue inhibitor of metalloproteinase 1 (TIMP1) | X03124 | 0.93 | 1.92E-03 | 1.83 | 0.15 | 1.16 |
| nucleoside diphosphate kinase B; NM23B | L16785 | 0.92 | 2.26E-03 | 1.57 | 6.8E-04 | 1.98 |
| follistatin-related protein | U06863 | 0.91 | 2.64E-03 | 0.38 | 9.9E-03 | 0.61 |
| purine-rich element-binding protein A (PURA) | M96684 | 0.90 | 2.80E-03 | 0.45 | 1.94E-02 | 0.64 |
| G1/S-specific cyclin D1 (CCND1) | X59798 | 0.90 | 3.02E-03 | 0.50 | 0.09 | 0.67 |
| MAP kinase-activated protein kinase 2 | U12779 | 0.89 | 3.28E-03 | 1.74 | 2.88E-03 | 1.86 |
| tumor necrosis factor receptor superfamily member 1B | M32315 | 0.89 | 3.28E-03 | 1.99 | 0.18 | 1.34 |
| procollagen IV alpha 2 subunit (COL4A2) | X05562 | 0.89 | 3.31E-03 | 0.54 | 3.3E-02 | 0.44 |
| FOS-related antigen 1 (FRA1) | X16707 | 0.88 | 3.62E-03 | 4.93 | 1.59E-02 | 3.63 |
| serine/threonine protein phosphatase alpha 1 catalytic subunit | M63960 | 0.88 | 3.73E-03 | 1.59 | 2.6E-02 | 1.36 |
