# Supplementary material for: Gene expression in acute Stanford type A dissection: a comparative microarray study
Source: J Transl Med. 2006 Jul 6;4:29. doi: 10.1186/1479-5876-4-29 (PMC1557406; doi:10.1186/1479-5876-4-29)
Supplement: Additional File 5 — Genes involved in discrimination between control and dissected aorta samples ordered by VIP as defined by SIMCA-P software using Cardiovascular arrays (Clontech platform). PLS-DA results are listed together with P values from t test for corresponding genes for Cardiovascular and Affymetrix arrays. [file 1479-5876-4-29-S5.doc]

**Supplemental Table 5. Genes involved in discrimination between control and dissected aorta samples ordered by *VIP* as defined by SIMCA-P software using Cardiovascular arrays (Clontech platform). PLS-DA results are listed together with *P* values from *t* test for corresponding genes for Cardiovascular and Affymetrix arrays.**

| Gene Name | GenBank | VIP | *P* value (*t* test) | ratio (D/C) | *P* value;  Affymetrix | ratio (D/C);  Affymetrix |
| --- | --- | --- | --- | --- | --- | --- |
| low-density lipoprotein receptor (LDLR) | M28219 | 1.18 | 7.42E-06 | 0.36 | 9.38E-03 | 1.61 |
| natriuretic peptide receptor A/guanylate cyclase A | X15357 | 1.18 | 8.56E-06 | 0.44 | 6.09E-05 | 0.29 |
| cardiotrophin 1 | U43030 | 1.15 | 2.95E-05 | 0.42 | 2.26E-02 | 0.5 |
| filamin A, alpha | X53416 | 1.14 | 3.70E-05 | 0.33 | 3.13E-04 | 0.48 |
| aquaporin 2 | S73197 | 1.10 | 1.08E-04 | 2.77 | No exp. | - |
| tissue inhibitor of metalloproteinase2 (TIMP2) | J05593 | 1.09 | 1.41E-04 | 0.41 | 0.086 | 0.76 |
| natriuretic peptide receptor C/guanylate cyclase C | X52282 | 1.08 | 2.00E-04 | 0.52 | 0.03 | 0.47 |
| (mast9; hevin) | X82157 | 1.06 | 2.96E-04 | 0.32 | 3.44E-06 | 0.61 |
| collagen, type XIV alpha 1 (undulin) | M64108 | 1.04 | 4.49E-04 | 0.43 | 1.42E-06 | 0.5 |
| melanoma cell adhesion molecule | M28882 | 1.02 | 6.34E-04 | 0.32 | 2.02E-04 | 0.47 |
| vinculin | M33308 | 1.02 | 7.29E-04 | 0.22 | 5.08E-04 | 0.61 |
| solute carrier family 4; anion exchanger, member 3 | L27213 | 1.01 | 8.20E-04 | 0.52 | No exp. | - |
| CD9 antigen (p24) | M38690 | 1.00 | 9.93E-04 | 0.55 | 7.45E-04 | 0.46 |
| tissue inhibitor of metalloproteinase 3 (TIMP3) | U14394 | 1.00 | 9.98E-04 | 0.24 | 0.33 | 0.72 |
| tissue inhibitor of metalloproteinase 1 (TIMP1) | X03124 | 0.99 | 1.07E-03 | 1.81 | 0.15 | 1.16 |
| annexin A3 | M20560 | 0.99 | 1.24E-03 | 0.17 | 5.92E-04 | 0.36 |
| sterol regulatory element binding transcription factor 2 | U02031 | 0.97 | 1.62E-03 | 0.44 | 0.4 | 0.9 |
| adrenergic alpha-1B- receptor | U03865 | 0.97 | 1.70E-03 | 0.58 | No exp. | - |
| superoxide dismutase 2; mitochondrial | M36693 | 0.97 | 1.71E-03 | 2.57 | 4.0E-04 | 3.82 |
| aldehyde dehydrogenase 2 family (mitochondrial) | X05409 | 0.96 | 1.96E-03 | 0.32 | 2.81E-03 | 0.57 |
| annexin A6 | D00510 | 0.95 | 2.13E-03 | 0.65 | 0.08 | 0.8 |
| hexabrachion (tenascin C; cytotactin) | X78565 | 0.90 | 4.62E-03 | 2.56 | 2.02E-03 | 6.22 |
| superoxide dismutase 3; extracellular | J02947 | 0.90 | 4.88E-03 | 0.44 | 1.57E-03 | 0.36 |
| galactosidase alpha | X05790 | 0.90 | 4.88E-03 | 0.43 | Abs. | - |
| P450 (cytochrome) oxidoreductase | S90469 | 0.89 | 5.30E-03 | 1.57 | 4.23E-03 | 1.38 |
| 3-oxoacid CoA transferase | U62961 | 0.88 | 5.78E-03 | 0.33 | 0.028 | 0.59 |
| glucosamine (N-acetyl)-6-sulfatase (Sanfilippo disease IIID) | Z12173 | 0.88 | 6.28E-03 | 0.66 | 0.43 | 1.19 |
| gastric inhibitory polypeptide | M18185 | 0.88 | 6.38E-03 | 0.62 | No exp. | - |
| endothelin 2 | M65199 | 0.88 | 6.47E-03 | 0.56 | No exp. | - |
| diaphorase (NADH) (cytochrome b-5 reductase) | Y09501 | 0.87 | 6.67E-03 | 0.52 | 0.2 | 0.86 |
