# Supplementary material for: Gene expression in acute Stanford type A dissection: a comparative microarray study
Source: J Transl Med. 2006 Jul 6;4:29. doi: 10.1186/1479-5876-4-29 (PMC1557406; doi:10.1186/1479-5876-4-29)
Supplement: Additional File 6 — Genes involved in discrimination between control and dissected aorta samples ordered by VIP as defined by SIMCA-P software using data from Affymetrix platform. PLS-DA results are listed together with P values from t test for corresponding genes for Affymetrix arrays. [file 1479-5876-4-29-S6.doc]

**Supplemental Table 6. Genes involved in discrimination between control and dissected aorta samples ordered by *VIP* as defined by SIMCA-P software using data from Affymetrix platform. PLS-DA results are listed together with *P* values from *t* test for corresponding genes for Affymetrix arrays.**

| Gene Name | Gene Symbol | Public ID | Gene Ontology Biological Process | ratio (D/C) | *P* value (*t* test) | *VIP* |
| --- | --- | --- | --- | --- | --- | --- |
| growth arrest-specific 6 | GAS6 | L13720 | cell adhesion; cell proliferation; regulation of cell growth | 0.28 | 3.52E-05 | 1.01 |
| prohibitin | PHB | AL560017 | DNA metabolism; cell growth and/or maintenance; negative regulation of cell proliferation/transcription | 2.67 | 5.79E-05 | 1.01 |
| peptidylprolyl isomerase F (cyclophilin F) | PPIF | BC005020 | protein folding | 5.29 | 3.16E-04 | 0.98 |
| Myosin, heavy polypeptide 11 | MYH11 | S67238 | cell growth and/or maintenance; muscle development | 0.32 | 8.33E-05 | 1.00 |
| non-metastatic cells 1, protein (NM23A) expressed in | NME1 | NM_000269 | CTP biosynthesis; GTP/UTP biosynthesis; negative regulation of cell cycle | 3.24 | 1.16E-04 | 1.00 |
| RuvB-like 1 (E, coli) | RUVBL1 | NM_003707 | DNA recombination; transcription | 2.86 | 1.49E-04 | 1.00 |
| caldesmon 1 | CALD1 | AL577531 | muscle contraction/development | 0.39 | 1.62E-04 | 0.99 |
| nucleoside phosphorylase | NP | NM_000270 | DNA modification; nucleobase, nucleoside, nucleotide and nucleic acid metabolism | 3.41 | 2.52E-04 | 0.99 |
| protein phosphatase 1; regulatory (inhibitor) subunit 12B | PPP1R12B | AF324888 | regulation of muscle contraction; signal transduction | 0.26 | 5.93E-05 | 1.01 |
| growth arrest-specific 6 | GAS6 | NM_000820 | cell adhesion; cell proliferation | 0.21 | 2.73E-05 | 1.01 |
| cysteine-rich motor neuron 1 | CRIM1 | BG546884 | neurogenesis | 0.25 | 2.26E-04 | 0.99 |
| solute carrier family 25, member 4 | SLC25A4 | NM_001151 | energy pathways | 0.37 | 4.19E-04 | 0.98 |
| alanyl (membrane) aminopeptidase (aminopeptidase N, CD13, p150) | ANPEP | NM_001150 | angiogenesis; proteolysis and peptidolysis | 7.51 | 2.03E-04 | 0.99 |
| inositol polyphosphate-5-phosphatase, 40kDa | INPP5A | NM_005539 | cell communication | 0.38 | 3.48E-04 | 0.98 |
| catenin, beta interacting protein 1 | CTNNBIP1 | NM_020248 | Wnt receptor signaling pathway; cell prolifer,; regulation of transcription | 0.38 | 2.69E-04 | 0.99 |
| Down syndrome critical region gene 1-like 1 | DSCR1L1 | NM_005822 | calcium-mediated signaling | 0.21 | 3.51E-05 | 1.01 |
| polycystic kidney disease 2 | PKD2 | NM_000297 | cation transport; cell-matrix adhesion | 0.37 | 2.19E-04 | 0.99 |
| frizzled-related protein | FRZB | NM_001463 | Wnt receptor signaling pathway; skeletal development | 0.38 | 1.48E-04 | 1.00 |
| adenosine deaminase | ADARB1 | NM_015833 | central nervous system development; mRNA processing | 0.22 | 4.17E-05 | 1.01 |
| dystrophin (muscular dystrophy, Duchenne and Becker types) | DMD | NM_004010 | cytoskeletal anchoring; muscle contraction | 0.35 | 3.51E-06 | 1.02 |
| phospholipase C, beta 4 | PLCB4 | AL535113 | intracellular signaling cascade; lipid catabolism/metabolism | 0.30 | 2.50E-04 | 0.99 |
| glutamate-cysteine ligase, modifier subunit | GCLM | NM_002061 | cysteine metabolism; glutathione biosynthesis | 3.38 | 1.36E-06 | 1.03 |
| proline arginine-rich end leucine-rich repeat protein | PRELP | NM_002725 | skeletal development | 0.20 | 1.97E-04 | 0.99 |
| leucine zipper, down-regulated in cancer 1 | LDOC1 | NM_012317 | negative regulation of cell proliferation | 0.36 | 1.73E-04 | 0.99 |
| GTP binding protein overexpressed in skeletal muscle | GEM | NM_005261 | cell growth and/or maintenance; small GTPase mediated signal transduction | 0.34 | 2.17E-04 | 0.99 |
| ankyrin repeat domain 6 | ANKRD6 | BE677131 | --- | 0.35 | 7.64E-05 | 1.00 |
| ankyrin repeat domain 6 | ANKRD6 | NM_014942 | --- | 0.32 | 9.21E-05 | 1.00 |
| phospholamban | PLN | NM_002667 | calcium ion transport; muscle contraction | 0.33 | 7.35E-05 | 1.00 |
| actin, alpha, cardiac muscle | ACTC | NM_005159 | muscle contraction; regulation of heart rate | 0.13 | 8.32E-06 | 1.02 |
| myomesin 1 (skelemin), 185kDa | MYOM1 | NM_003803 | muscle development; striated muscle contraction | 0.33 | 1.60E-04 | 0.99 |
| transient receptor potential cation channel, subfamily C, member 1 | TRPC1 | NM_003304 | calcium ion transport; cation transport | 0.30 | 2.35E-05 | 1.01 |
| osteomodulin | OMD | AI765819 | cell adhesion | 0.28 | 2.83E-04 | 0.99 |
| extracellular matrix protein 2, female organ and adipocyte specific | ECM2 | NM_001393 | cell-matrix adhesion | 0.29 | 3.39E-04 | 0.98 |
| tropomyosin 1 (alpha) | TPM1 | NM_000366 | muscle development/contraction | 0.36 | 2.36E-04 | 0.99 |
| melanoma cell adhesion molecule | MCAM | BE964361 | cell adhesion | 0.35 | 1.90E-04 | 0.99 |
| spectrin repeat containing, nuclear envelope 1 | SYNE1 | AF043290 | Golgi organization and biogenesis; muscle cell differentiation | 0.40 | 3.28E-04 | 0.98 |
| carboxylesterase 1 (serine esterase 1) | CES1 | S73751 | metabolism; response to toxin | 0.28 | 3.60E-04 | 0.98 |
| transmembrane 4 superfamily member 10 | TM4SF10 | AI803181 | --- | 0.24 | 3.75E-04 | 0.98 |
| transmembrane 4 superfamily member 10 | TM4SF10 | AL136550 | --- | 0.34 | 5.55E-05 | 1.01 |
| uridine-cytidine kinase 2 | UCK2 | BC002906 | biosynthesis | 2.70 | 1.41E-04 | 1.00 |
| BH-protocadherin (brain-heart) | PCDH7 | AB006757 | cell adhesion | 0.37 | 2.02E-04 | 0.99 |
| serine protease inhibitor, Kunitz type 2 | SPINT2 | AF027205 | cell motility | 0.30 | 4.14E-05 | 1.01 |
| melanoma cell adhesion molecule | MCAM | M29277 | cell adhesion | 0.39 | 4.92E-05 | 1.01 |
| contactin 1 | CNTN1 | U07820 | cell adhesion | 0.38 | 8.10E-06 | 1.02 |
| KIAA1102 protein | KIAA1102 | AK026815 | smooth muscle contraction | 0.39 | 1.01E-04 | 1.00 |
| myosin, heavy polypeptide 10 | MYH10 | AK026977 | cellular morphogenesis; cytokinesis | 0.35 | 3.42E-06 | 1.02 |
| desmuslin | DMN | AK026420 | --- | 0.37 | 2.33E-05 | 1.01 |
| sortilin 1 | SORT1 | BF447105 | endocytosis; intracellular protein transport | 0.37 | 2.28E-04 | 0.99 |
| sterile alpha motif domain cont, 4 | SAMD4 | AB028976 | --- | 0.36 | 9.31E-05 | 1.00 |
| chromobox homolog 7 | CBX7 | AV648364 | chromatin assembly or disassembly; regulation of transcription | 0.32 | 7.06E-05 | 1.00 |
| phosphodiesterase 8B | PDE8B | AK023913 | cyclic nucleotide metabolism; signal transduction | 0.39 | 2.67E-04 | 0.99 |
| ADAMTS-like 3 | ADAMTSL3 | AB033059 | --- | 0.30 | 4.96E-05 | 1.01 |
| hypothetical protein HSPC111 | HSPC111 | BE314601 | --- | 2.66 | 2.44E-04 | 0.99 |
| complement component 1, q subcomponent binding protein | C1QBP | AU151801 | immune response | 2.99 | 1.43E-06 | 1.03 |
| integrin, alpha 8 | ITGA8 | AI193623 | cell-cell adhesion; cell-matrix adhesion; integrin-mediated signaling | 0.24 | 6.35E-06 | 1.02 |
| nephroblastoma overexpressed gene | NOV | BF440025 | regulation of cell growth | 0.29 | 4.67E-05 | 1.01 |
| collagen, type VIII, alpha 1 | COL8A1 | BE877796 | cell adhesion; phosphate transport | 0.32 | 8.61E-05 | 1.00 |
| tryptophan hydroxylase 1 | TPH1 | AI350339 | metabolism | 0.25 | 3.35E-06 | 1.02 |
| --- | --- | AL359052 | --- | 0.24 | 3.36E-04 | 0.98 |
| KIAA0527 protein | KIAA0527 | BF977837 | --- | 0.33 | 7.45E-07 | 1.03 |
| KIAA0774 | KIAA0774 | AI818409 | --- | 0.32 | 3.14E-04 | 0.98 |
| superoxide dismutase 2, mitochondrial | SOD2 | W46388 | age-dependent response to reactive oxygen species; cellular defense response | 3.82 | 4.00E-04 | 0.98 |
| LIM domain binding 3 | LDB3 | AJ133768 | --- | 0.17 | 1.67E-04 | 0.99 |
| dystrophia myotonica-protein kinase | DMPK | M87313 | muscle contraction | 0.20 | 2.49E-04 | 0.99 |
| basic leucine zipper and W2 domains 2 | BZW2 | NM_014038 | regulation of translational initiation | 2.84 | 1.16E-05 | 1.02 |
| synaptotagmin binding | SYNCRIP | NM_006372 | RNA splicing | 3.22 | 2.55E-06 | 1.03 |
| --- | --- | NM_024658 | --- | 3.40 | 3.32E-05 | 1.01 |
| Chr, 10 open reading frame 70 | C10orf70 | NM_018464 | --- | 2.53 | 2.50E-05 | 1.01 |
| asporin (LRR class 1) | ASPN | NM_017680 | --- | 0.21 | 1.28E-05 | 1.02 |
| solute carrier family 24, member 3 | SLC24A3 | NM_020689 | calcium/potassium ion transport | 0.30 | 4.05E-04 | 0.98 |
| lipase, endothelial | LIPG | NM_006033 | lipid catabolism | 3.47 | 4.07E-04 | 0.98 |
| platelet derived growth factor D | PDGFD | NM_025208 | cell growth and/or maintenance | 0.37 | 6.43E-05 | 1.01 |
| protein tyrosine phosphatase-like member a | PTPLA | NM_014241 | --- | 0.34 | 4.19E-05 | 1.01 |
| likely ortholog of mouse limb-bud and heart gene | LBH | NM_030915 | --- | 0.29 | 1.19E-04 | 1.00 |
| natriuretic peptide receptor A/guanylate cyclase A | NPR1 | X15357 | cGMP biosynthesis; regulation of blood pressure/vascular permeability | 0.29 | 6.09E-05 | 1.01 |
| thromboxane A2 receptor | TBXA2R | D38081 | G-protein coupled receptor protein signaling pathway; muscle contraction | 0.37 | 3.35E-04 | 0.98 |
| dystrophia myotonica-protein kinase | DMPK | L08835 | muscle contraction | 0.29 | 1.20E-04 | 1.00 |
| chondroitin sulfate proteoglycan 5 (neuroglycan C) | CSPG5 | AF059274 | intracellular transport; neurogenesis | 0.26 | 2.26E-04 | 0.99 |
| host cell factor C1 regulator 1 | HCFC1R1 | AA436930 | --- | 0.37 | 4.15E-05 | 1.01 |
