# Supplementary material for: Gene expression in acute Stanford type A dissection: a comparative microarray study
Source: J Transl Med. 2006 Jul 6;4:29. doi: 10.1186/1479-5876-4-29 (PMC1557406; doi:10.1186/1479-5876-4-29)

A

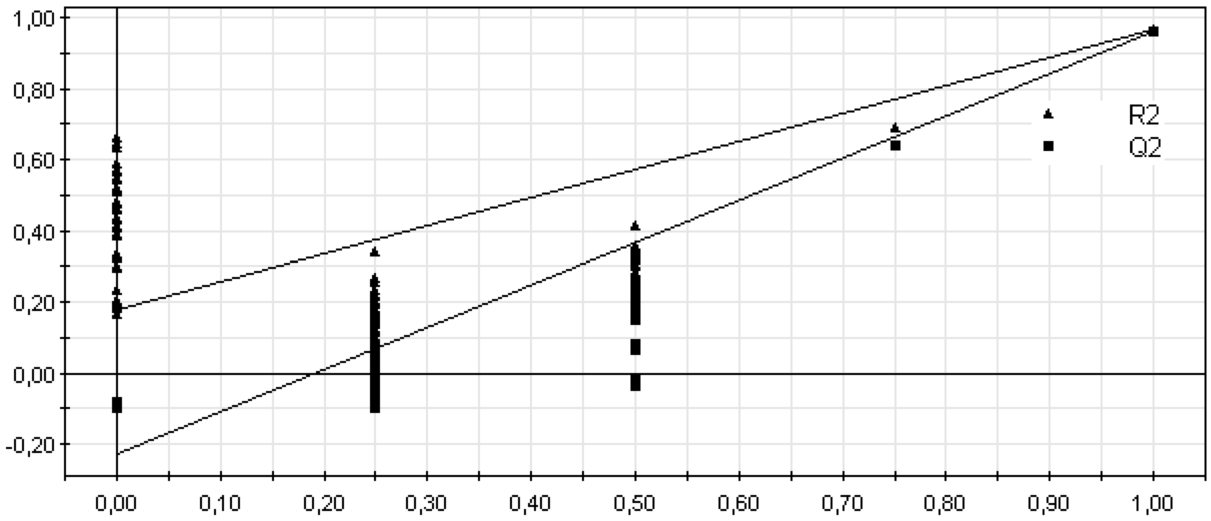

B

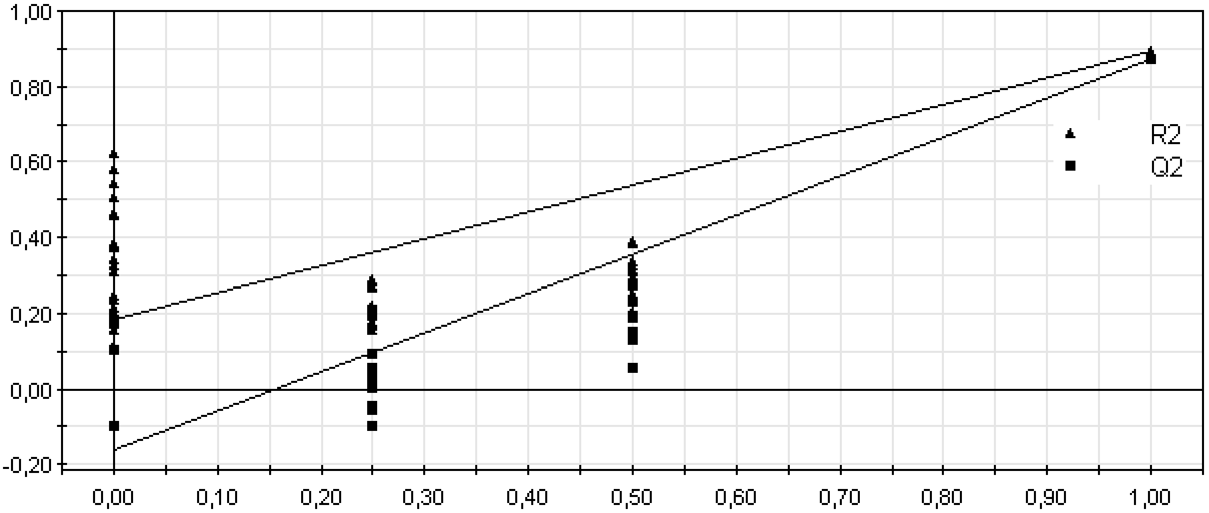

50 permutations 1 components

C

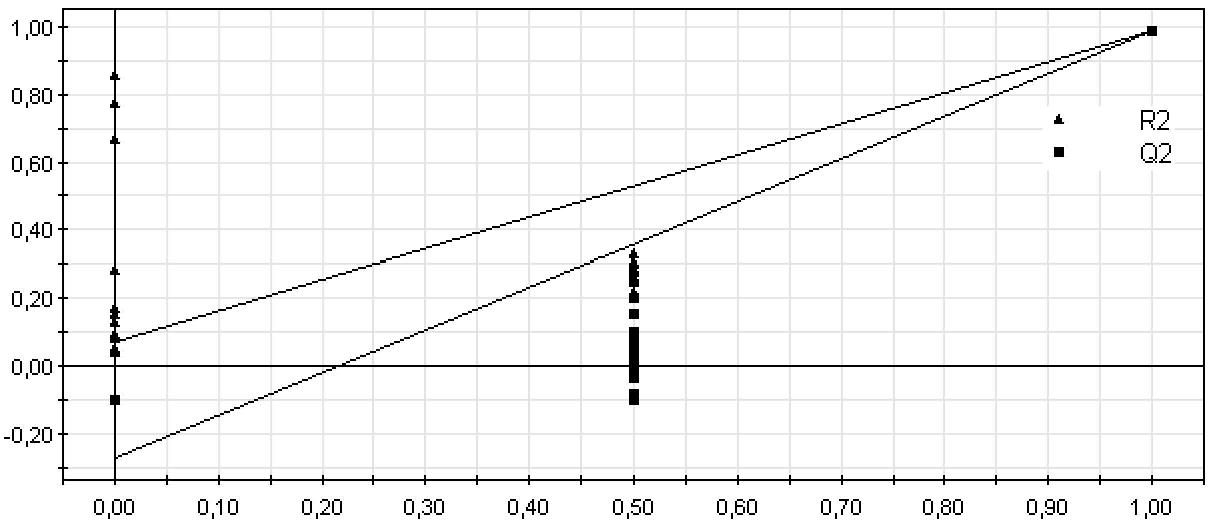

Supplement: Supplementary file 7 [file 1479-5876-4-29-S7.doc]
